# Enhancing arthropod occurrence in wheat cropping systems: the role of non-chemical pest management and nitrogen optimization

**DOI:** 10.1007/s10661-024-12709-9

**Published:** 2024-05-23

**Authors:** Julia Gitzel, Helge Kampen, Jörg Sellmann, Jürgen Schwarz, Luca Marie Hoffmann, Stefan Kühne, Christian Ulrichs, Doreen Werner

**Affiliations:** 1 Leibniz Centre for Agricultural Landscape Research, Müncheberg, Germany; 2 Friedrich-Loeffler-Institut, Federal Research Institute for Animal Health, Greifswald, Germany; 3 Humboldt-Universität zu Berlin, Thaer-Institute, Urban Plant Ecophysiology, Berlin, Germany; 4 Institute for Strategies and Technology Assessment, Julius Kühn-Institut, Kleinmachnow, Germany

**Keywords:** Arthropod monitoring, Arthropod biomass, Biodiversity, Sweeping nets, Eclector traps, Yellow trap

## Abstract

This study analyzes arthropod biomass and abundance to track the changes in arthropod occurrence in relation to pesticide use in three winter wheat cropping systems managed at different intensities (organic, conventional, and hybrid). Arthropod occurrence was surveyed using three collection tools: sweeping nets, eclector traps, and yellow traps. Sampling was conducted over three years from 2020 to 2022 with 588 samples collected. The wet weight of the captured organisms was determined and arthropod abundance calculated. The application of a NOcsPS (no chemical-synthetic pesticides) strategy, a new hybrid cultivation method realized with optimized use of nitrogen fertilizers but without chemical-synthetic pesticides, showed a higher arthropod occurrence and performed more convincingly regarding produced arthropod biomass and abundance than the other cropping variants. The results also demonstrate a dependence of the obtained insect indices on the collection method. Although arthropod biomass and abundance correlated for all collection methods, the combination of various methods as well as multiple procedures of sample analysis gives a more realistic and comprehensive view of the impact of the wheat cultivation systems on the arthropod fauna than one-factor analyses.

**Supplementary Information:**

The online version contains supplementary material available at 10.1007/s10661-024-12709-9.

## Introduction

After insect mortality has become a much-discussed topic in society, the media, and politics, the awareness of the important function of arthropods in nature is growing (Zhang, 2013; Crespo-Pérez et al., 2020). However, although insects have been identified to play important roles in ecosystems and agricultural production, e.g., as pollinators and natural pest controllers (Öckinger and Smith, 2007), the loss of insect biodiversity seems to continue and even to accelerate on a worldwide scale (Sánchez-Bayo & Wyckhuys, 2019). On top of that, current data suggest a decline in insect abundance (Hendrickx et al., 2007; Hallmann et al., 2017; Zhou et al., 2023). While the use of pesticides and habitat destruction are mainly made responsible for these developments, crop management and intensive land use have been identified as two of multiple contributing factors (Habel et al., 2019). In recent decades, the use of chemical-synthetic pesticides has increased worldwide, which has caused an increasing pressure on ecosystems (Tang et al., 2021).

Arthropods are not only sensitive to short-term impacts on agriculture, but also to long-term ecosystem changes (Kremen et al., 1993; Underwood & Fisher, 2006). During the last decades, many studies dealt with arthropod diversity and abundance in agricultural landscapes, focusing on various taxa and comparing either the current situation with previous conditions or different cultivation forms (Wagner et al., 2021). Consequently, a wide range of factors has been described that have an influence on arthropod diversity as well as on the biodiversity of fauna and flora in general. In addition to pesticide use, parameters such as fertilization, soil cultivation, crop rotation design, but also small structures adjacent to the production area (hedges, fringe biotopes) play important roles for biodiversity (Freier et al., 2017). Moreover, the various factors influence each other, and their impact is difficult to separate under field conditions. The control of insect pests by insecticides and of weeds by herbicides or mechanical approaches also directly or indirectly affect beneficial arthropods (Brühl and Zaller, 2019; Brühl et al., 2021). Indirect impact on non-target organisms is mainly expressed by deprivation of food sources and habitat loss (Freier et al., 2017).

In addition to the use of chemical-synthetic pesticides, the extensive application of mineral fertilizers, such as nitrogen, contributes to the loss of species, especially in conventional agriculture systems (Stevens et al., 2004; Martin & Sauerborn, 2006). Arthropods are particularly affected by the changing composition of weeds due to high nitrogen supply (David et al., 2019). The application of herbicides and intensive soil cultivation not only have a negative impact on flowering weeds and beneficial insects, but also on predator-prey relationships (Krauss et al., 2011). All these factors alone, and the combination of them, lead to reductions in arthropod species and abundance (Wagner et al., 2021).

A widely adopted method for examining the presence and activity patterns of arthropods involves assessing their biomass. This approach, exemplified by the Krefeld study (Hallmann et al., 2017), assumes that biomass offers a more accurate reflection of insect status and ecosystem contributions compared to deducing the overall biodiversity impact from different insect taxa (Sorg et al. 2013). Thus, insect biomass is often used as an indicator for the measurement of ecosystem function (Dangles et al., 2011; Barnes et al., 2016). However, it is argued that high biomass is not necessarily an indicator of high abundance, physical size, and species diversity (Welti et al., 2021). As a measure of insect occurrence, other studies only assess the insect abundance or species richness of different families (Homburg et al., 2019; Bengtsson et al., 2005).

To reliably determine the status of a particular group of insects in a certain habitat, the selection of appropriate determination approaches is most important (Russo et al., 2011). Depending on the taxa to be captured, different collection methods are recommended (Missa et al., 2009; Joshi et al., 2015; Montgomery et al., 2021). To obtain a fully comprehensive picture of insect biodiversity, biomass, and abundance in a habitat, it appears advisable to combine several methods such as sweeping nets, yellow traps, and eclector traps.

The sweeping net method involves sweeping through vegetation with a wide net to capture flying and jumping arthropods in upper vegetation levels. This method is effective in capturing a wide variety of arthropods, especially those that are active during the day and in open areas. However, it may miss ground-dwelling or nocturnal species (Zou et al., 2012; Spafford & Lortie, 2013). Netting proves to be particularly suitable for comparing abundance and richness of small arthropods occurring in similar vegetation types (Evans et al., 1983). By contrast, yellow traps are passive collection devices that attract arthropods with their bright yellow color (Campbell & Hanula, 2007; Abrahamczyk et al., 2010). They are particularly effective for capturing flying insects such as aphids, thrips, and leafhoppers. However, they may not be as efficient in capturing non-flying arthropods or arthropods not attracted to the color yellow. Eclector traps are designed to capture arthropods that inhabit the soil and hatch from soil, providing valuable information about subterranean arthropod populations. Eclector traps show promising potential for investigating invertebrates within agroecosystems. They complement pitfall traps by offering direct density estimates within a confined area (McCravy, 2018). Based on three differently managed winter wheat cropping variants, the purpose of the present study was to determine, whether (i) the cropping variant influences arthropod biomass and abundance, (ii) there is a correlation between arthropod biomass and abundance, and (iii) the combination of arthropod biomass and abundance data gives a more comprehensive view of the arthropod occurrence present.

To answer these questions, three different entomological methods were used to collect and analyze arthropod occurrence.

## Materials and methods

### Study area and agricultural management

The study was conducted on the Julius Kühn-Institut experimental field area in Dahnsdorf, federal state of Brandenburg, Germany (N52.108494, E12.636338) from 2020 to 2022. The whole field, which covers 38 ha, is located at 77 to 85 m above sea level. Measurements for the period 1997–2021 produced a mean annual precipitation of 571 mm and a mean annual temperature of 9.6° C. The soil has a mean quality of 48 soil points according to the German soil classification (Adhoc-Arbeitsgruppe Boden, 2005) and consists of sandy loess (sand 57.9%, silt 37.5%, clay 4.6%, organic matter 1.4%), pH 5.8.

Arthropods were collected in three winter wheat cropping variants investigated under different levels of management: conventional, organic, and the new hybrid variant NOcsPS (NO chemical-synthetic pesticides).

The experiment was based on a six-year crop rotation design: winter wheat I (variety: Achim) - silage maize - winter rye - pea - winter wheat II (variety: RGT Reform) - spring barley. In the organic cropping variant, spring barley was replaced with clover grass to promote nitrogen fixation by bacteria in the root follicles, as there was no additional nitrogen fertilization. All variants had been applied on the wheat variety “RGT Reform.” In 2020, the organic cropping variant was drilled in the variety “Govelino,” while in the following years, the wheat variety “RGT Reform” was chosen to exclude an impact of variety differences, such as resistance to various fungi and yield differences.

**Fig. 1:**
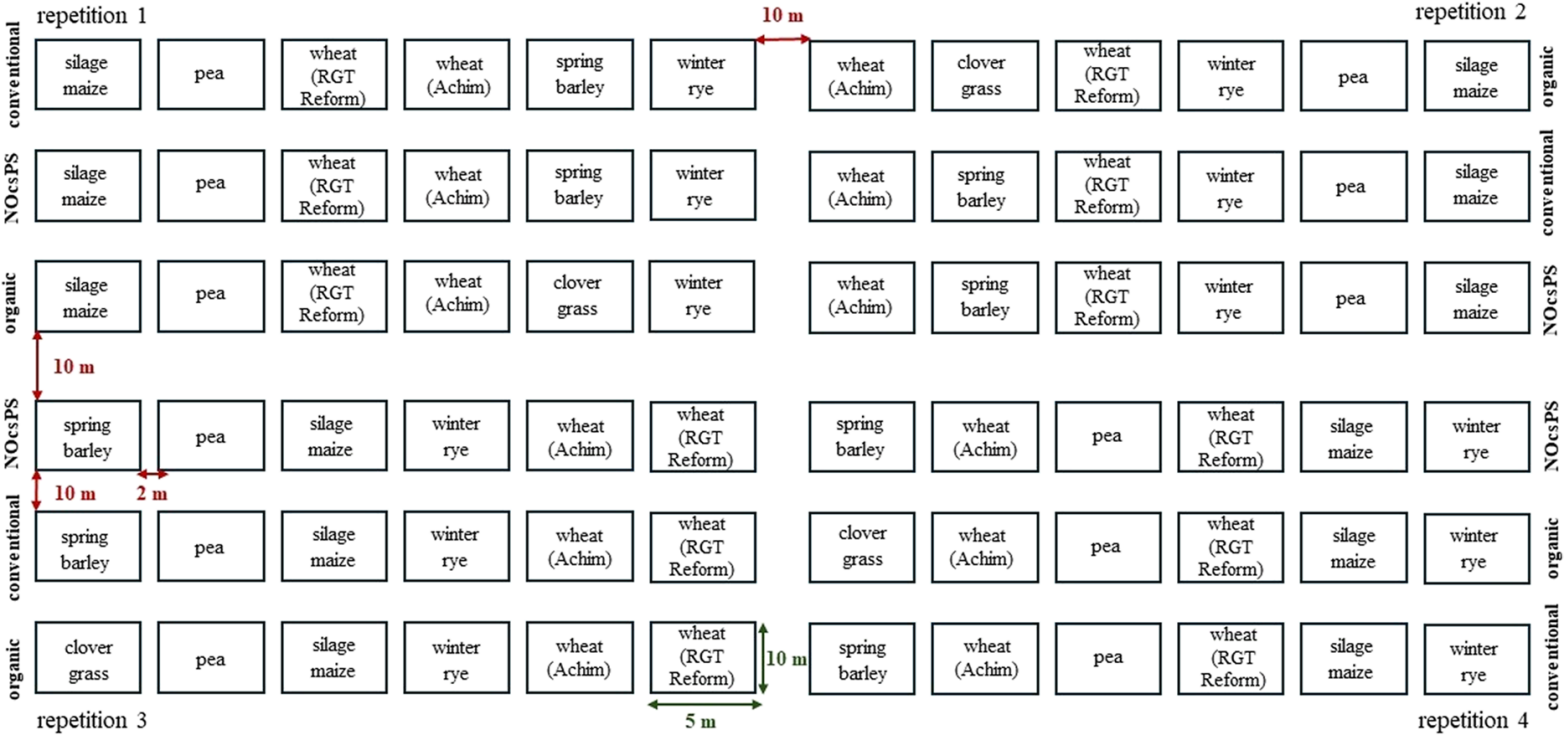
Experimental design of the six-year crop rotation trial in 2022 for the cropping variants organic, NOcsPS, and conventional

Each crop was grown in four repetitions, and each repetition contained four plots of each crop in the study field. The size of each experimental plot was 5 m x 10 m (50 m^2^). In total, 12 plots were sampled (four per cropping variant) (Fig. 1). The study was carried out in a randomized block design. This resulted in different spatial positions of the crop variant plots in the repetitions, minimizing edge effects and differences in soil fertility and water-holding capacity. The position of the crop in the repetitions changed every year. Soil samples were taken to analyze the mineral composition of the soil and, in the case of conventional and NOcsPS cropping variants, to adjust fertilization according to the site.

The conventional cropping variant was drilled with 360 grains/m^2^. The working width of the seed drill was 2 x 2.5 m. Drilling was carried out with 12.5 cm spacings between the rows. The drilling scheme was as follows: three rows of seed, two empty rows (lane), ten rows of seed, two empty rows (lane), six rows of seed, two empty rows (lane), ten rows of seed, two empty rows (lane), and three rows of seed. Seed grains were pickled (Landor CT in 2020 (composition: fludioxonil 25 g/l (2.4% by weight), difenoconazole 20 g/l (1.9 wt.%), tebuconazol 5 g/l (0.5 wt.%)) and Rubin TT in 2021 and 2022 (composition: prochloraz 38.6g/l (42g/l copper chloride complex), pyrimethanil 42g/l, triticonazole 25g/l). The agricultural process involved initial soil cultivation through plowing, succeeded by the precise distribution of wheat seeds utilizing a rotary harrow in conjunction with a seed drill. Soil cultivation, seeding, and mechanical weeding were carried out in the plot (Supplementary Table S1). Fertilization and plant protection were managed from the outside, i.e., the machines did not drive through the crop. Nitrogen fertilization took place with KAS 27 % N (calcium ammonium salts with 13.5 % nitrate nitrogen and 13.5 % ammonium nitrogen) and pure nitrogen every year in March (370 kg/ha KAS and 100 kg/ha N in 2020 and 2021, 296 kg/ha KAS and 79.9 kg/ha N in 2022). Additional nitrogen was added at the end of April (148 kg/ha KAS and 40 kg N/ha in 2020 and 2021, 111 kg/ha KAS and 30 kg N/ha in 2022). Additional fertilization protocols are detailed in Supplementary Table S1. This variant was managed according to integrated crop production. Pesticides were only applied after the pest thresholds had been exceeded (according to weekly pest assessments and monitoring). This approach resulted in an application of insecticides in 2021 and 2022: in November 2021 (BBCH 12/13), the larvae of ground beetle (*Zabrus tenebrioides* (Goeze, 1777)) were controlled with the insecticide Karate Zeon (75 ml/ha, 100 g/l active ingredient lambda-cyhalothrin) in repetitions 1 and 4, but not in repetitions 2 and 3, in which the pest thresholds had not yet been exceeded. The treatment was also carried out in March 2022 (BBCH 23) in repetition 2, after surpassing the thresholds. Herbicides were applied after germination of the wheat. In 2019, 1 l/ha of the herbicide Bacara Forte (active ingredient 120 g/l diflufenican, 120 g/l flufenacet, 120 g/l flurtamone) was applied once in BBCH 13. In 2020, 1.5 l/ha of the herbicide Trinity (active ingredient 300 g/l pendimethalin, 250 g/l chlortoluron, 40 g/l diflufenican) was brought out once in BBCH 12. Trinity was also applied in fall 2021, with an application volume of 2.0 l/ha. In 2020 and 2021, treatment against the fungus *Septoria tritici* was necessary. Application of 1.25 l/ha Input Classic fungicide (active ingredient 160 g/l prothioconazole, 300 g/l spiroxamine) was realized in BBCH 37 in 2020 and a combination of 1.2 l/ha Revytrex (active ingredient 66.7 g/l fluxapyroxad, 66.7 g/l mefentrifluconazole) with 0.4 l/ha Comet (active ingredient 200 g/l pyraclostrobin) in BBCH 43 in 2021 (Supplementary Table S1).

The organic cropping variant was managed according to the Regulation (EU) 2018/848 and its corresponding implementing regulations (The European Parliament and the Council of the European Union, 2018). This variant was drilled with 380 grains/m^2^ (every year in the first week of October). The working width of the seed drill was 2 x 2.5 m. The plots were managed like the conventional cropping variant (Supplementary Table S1). One row of drills had a spacing of 12.5 cm, and the drill scheme was also similar to the conventional cropping variant. In the organic cropping variant, enough nitrogen is bound in the soil by the green manures in the form of pea (previous crop) and clover grass (post crop). Further fertilization measures realized are depicted in Supplementary Table S1. Weekly monitoring and assessments for pests were conducted; however, due to unavailability of organic pesticides effective against aphids, fungi, and ground beetles, no treatments were administered. Chemical-synthetic insecticides and fungicides were not used. Furthermore, the use of herbicides was dispensed with. Mechanical weed control took place in form of weeding once in fall in 2021 and 2022 (before germination) and in spring of every study year (BBCH 22-25) (Supplementary Table S1).

The new hybrid cropping variant NOcsPS was drilled in single grain placement, which resulted in 250 drilled grains/m^2^. Therefore, the row width amounted to 15 cm, instead of 12.5 cm. The working width of the seed drill were 3 m and 1.95 m. This variant was therefore 5 cm shorter than the conventional and the organic cropping variant. The excess rows were routed. The drill scheme was as follows: two rows of seed, two empty rows (lane), eight rows of seed, two empty rows (lane), five rows of seed, two empty rows (lane), eight rows of seed, two empty rows (lane) and two rows of seed. Adapted nitrogen fertilization was used (30% less amount compared to the conventional variant). Nitrogen application was as follows: 260 kg/ha KAS and 70 kg/ha N in March and 104 kg/ha KAS and 28 kg/ha N in April 2020; 185 kg/ha KAS and 50 kg/ha N in early March and 88 kg/ha KAS and 23.8 kg/ha N each in late March and late April 2021; 207 kg/ha KAS and 55.9 kg/ha N in late March and 44 kg/ha KAS and 11.9 kg/ha N in late April 2022. Additional fertilization implemented is detailed in Supplementary Table S1. As in the organic cropping variant, chemical-synthetic insecticides, fungicides, and herbicides were not applied. Weekly pest monitoring was conducted and documented but no pesticide treatment was carried out. Because of the avoidance of herbicides, mechanical weed control was adapted to the organic cropping variant and took place in the form of weeding once in fall in 2021 and 2022 (before germination) and in spring in every year (BBCH22-25) (Supplementary Table S1).

### Arthropod collection

Sweeping nets, eclector traps, and yellow traps were used to analyze arthropod biomass and abundance. Sampling with sweeping nets and eclector traps took place from early May to late June 2020 to 2022; yellow traps were only established in June of each year. All samples obtained by the three catching methods were collected on the same day of the week. The order in which the plots were sampled was randomized weekly.

Sweeping net catches were carried out on one day per week from the first week of May through the end of June. The collections took place between 10 a.m. and 1 p.m., when the flight activity of insects was at its peak, and only on dry days or within dry periods. The sweeping net had a diameter of 0.3 m and a handle length of 0.65 m. One catch consisted of 25 double scoops performed while walking through the lanes and sweeping the left and right rows (organic and conventional: row numbers 16/17 and row numbers 24/25; NOcsPS: row numbers 13/14 and row numbers 18/19). The sweeping net was always operated directly above the wheat stand. During the sampling dates in June, the top of the wheat stand was striped to capture arthropods that were attached to the spikes and flag leaves of the wheat. Depending on size and mobility, arthropods were removed from the net by means of an aspirator or transferred manually into 50 ml PE bottles containing 70% ethanol.

The used eclector traps had a footprint of 0.25 m^2^. They were positioned 2 m from the edge of the plots in the first week of May and moved further into the plot by approximately 1 meter every 14 days until the end of June. During this movement, the wheat stalks were shortened on the new stand (for about 10–15 cm) when reaching the height of the eclector trap. The cut stalks were placed under the trap to change the conditions for the flying and climbing arthropods as little as possible and to capture the arthropods attached to the stalks as well. The head containers of the traps were filled with a 1:1-mixture of tap water and ethylene glycol (70 ml of trapping liquid per trap). In addition, a drop of detergent was added to reduce surface tension. The impact on ethylene glycol on the attraction or repulsion remains largely unexplored. Previous own research conducted in 2019 (unpublished) demonstrated that ethylene glycol effectively preserves specimens when traps are emptied biweekly. Moreover, initial findings suggest that traps treated with ethylene glycol exhibit superior preservation of arthropods compared to control traps lacking the substance. The eclector traps were emptied every week on the same day.

The yellow traps (diameter: 25 cm) were placed on the ground in the second lane of each study plot so to be uncovered by surrounding vegetation (row numbers 16/17, 1.87 m from the edge, 2 m from the beginning of the lane in the organic and conventional cropping variants; row numbers 14/15, 1.95 m from edge, 2 m within the lane for NOcsPS cropping variant). They were positioned during the first week of June and remained there until the end of June. The trapping liquid consisted of a 1:4-mixture of water and ethylene glycol, with a drop of detergent to eliminate surface tension. The yellow traps were emptied every seven days, synchronized with the eclector traps and sweeping net operations.

### Calculation of arthropod biomass

All samples obtained with the three catching methods were preserved in 70% ethanol. To weigh their biomass, they were cleaned of impurities, such as small soil crumbs or plant parts. Samples were transferred onto a fine-mesh sieve (approx. 0.5 mm mesh size) and allowed to dry for a few seconds. The sieve was briefly pressed onto an absorbent paper towel and then tapped on a glass petri dish of a standardized tare weight. Small arthropods were transferred with a fine brush to avoid the loss of body parts. Biomass was then measured by weighing the sample with a fine balance scale (Sartorius AG Göttingen, model CP124S-0CE) with four decimal places. The value was read 10 s after setting up.

Biomass of the arthropod collections was measured for each cropping variant at each catch date. The collected data were recorded and mean and median values of all catching dates were calculated for each cropping variant according to the catching method (total arthropod biomass (of all plots) divided by number of catches). Data were prepared in ORACLE for statistical analysis using SAS.

### Calculation of arthropod abundance

To calculate the arthropod abundance, individuals were counted, categorized into insects and arachnids according to cropping and collection system, and organized chronologically by catching date. Furthermore, each field plot was analyzed individually. After the complete counting of the collected individuals, the mean and median values of all catching dates were calculated for each cropping system according to the catching method using SAS (total number of individuals (of all plots) divided by the number of catches).

The total arthropod population from the upper vegetation layer of wheat was calculated on the basis of the sweeping net catches. The yellow trap catches were used to determine the number of individuals and the activity of arthropods, especially pests, flying in the crop whereas the elector data were used to provide information about arthropods hatching from the soil.

### Arthropod richness

After weighing the arthropod biomass, the collected samples were taxonomically determined to order, and in certain instances, to family level (Supplementary Table S4), using identification keys of Müller and Bährmann (2015). Given the impracticality of conducting a taxonomic assessment at the species level with the high number of arthropods collected (Table 1), the statistical evaluation of the collected arthropods was made on order level. This approach aimed to demonstrate differences in arthropod richness among the different cropping variants. Each field plot underwent individual analysis, and the mean and median values of all capture dates were computed for each cropping variant, based on the catching method, using SAS software. This involved dividing the total number of orders (across all plots) by the number of catches.

**Table 1:** Number of collected insect and arachnid specimens according to study year, trapping method, and cropping variant

Cropping variant	Study period	Collection method					
		Eclector		Sweeping net		Yellow trap	
		Arachnida	Insecta	Arachnida	Insecta	Arachnida	Insecta
NOcsPS	2020	27	5,200	171	5,805	14	2,218
	2021	65	11,535	221	4,690	39	2,642
	2022	202	7,865	251	3,321	116	4,410
	Total	294	24,600	643	13,816	169	9,270
Conventional	2020	32	3,660	171	4,438	14	1,696
	2021	47	10,794	213	2,456	46	2,860
	2022	149	5,633	247	2,172	70	3,908
	Total	228	20,087	631	9,066	130	8,464
Organic	2020	18	4,972	156	4,865	21	1,387
	2021	101	6,977	193	2,613	33	2,137
	2022	135	5,274	246	4,108	74	3,507
	Total	254	17,223	595	11,586	128	7,031
Total over 3 years of trial)		776	61,910	1,869	34,468	427	24,765
Total of Arachnida							3,072
Total of Insecta							121,143

A summary of the arthropod orders, and in certain instances, families, and their abundances captured in the three catching methods is presented in Supplementary Table S4.

### Statistics

The program SAS 9.4 (SAS, Cary, NC, USA) was used to analyze and compare arthropod data collected from the plots with the various cropping variants. The results of partial effects of cropping systems on arthropod biomass, abundance, and richness were computed with linear models in case of biomass and generalized linear models regarding abundance and richness. The exponential family as a foundational framework was employed. For this purpose, the procedure glimmix with the specification dist=Poi distribution was used in SAS for arthropod richness and abundance (as count data). The Gaussian distribution was used for arthropod biomass. F and df values (collection date, cropping variant) as well as* p*-values were calculated for the predictors. The values are based on comparisons between organic, conventional, and NOcsPS cropping variants. The F-test was used to examine whether at least one of the predictors in the model was significant. For the post-hoc pairwise comparisons with the Tukey test, the key figures for the model, R^2^ (the coefficient of determination) and the *p*-value of the F-test were used. Linear regression analyses were used to describe the overall trends in arthropod biomass and abundance. A correlation analysis, including linear regression functions, was performed to relate arthropod biomass to arthropod abundance.

Bi-plots were used for presenting the results, which is a technique for visually presenting both the entities and the influential factors within a single plane. When clear correlations exist, the impact of individual factors, such as the management type in this context, on the multidimensional entities can be inferred from the graph. In our study, the three dimensions encompass arthropod biomass, abundance, and richness. Principal component analysis (PCA), also carried out using SAS with the multidimensional preference analysis (proc prinqual plots=MDP) specification, was conducted separately for three distinct catching methods.

## Results

### Arthropod biomass

Throughout the three years of the study, a total of 588 samples were collected. Hundred and forty-four samples each resulted from both the yellow trap and from the eclector trap collections. The sweeping net method produced 300 samples.

**Fig. 2:**
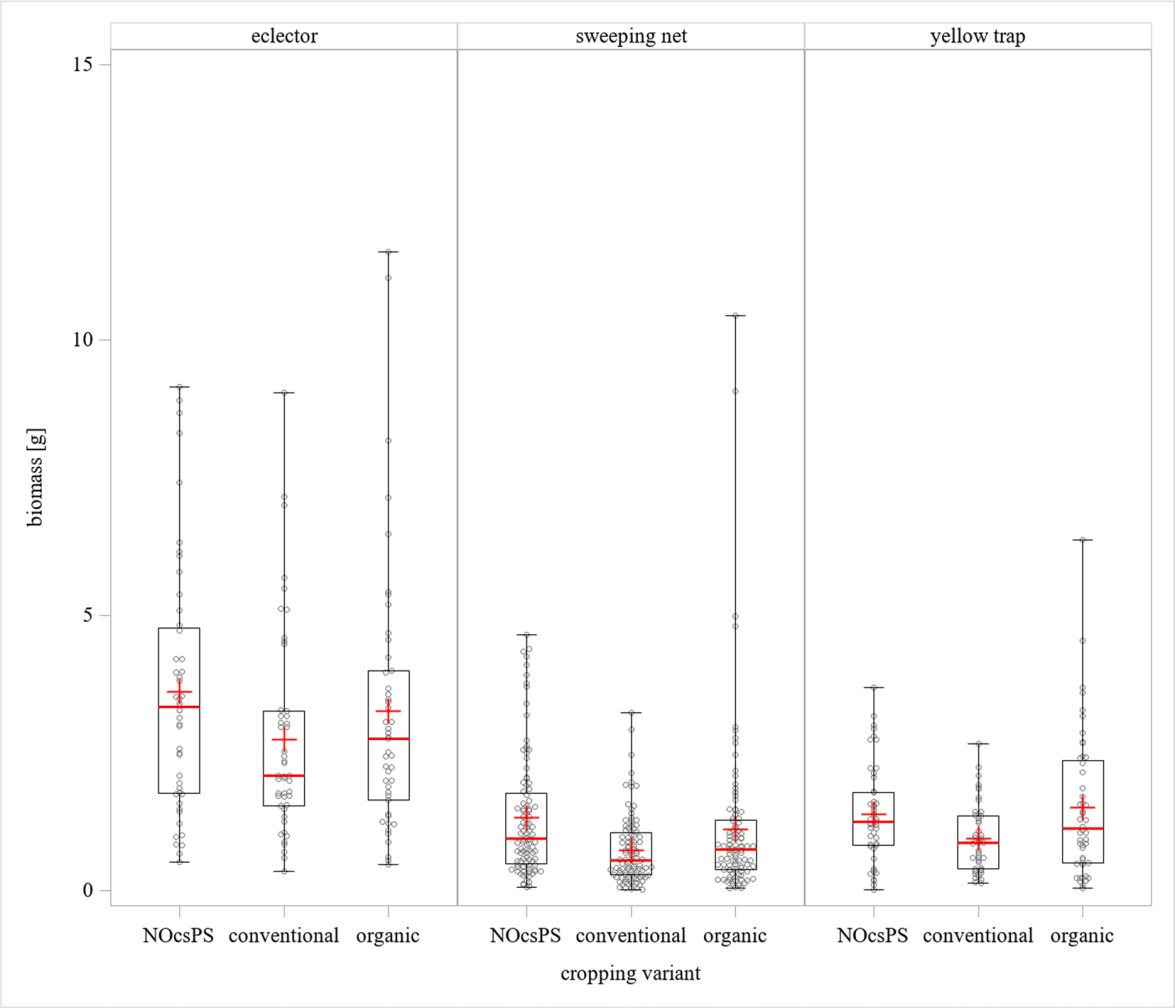
Partial effects of cropping variant on arthropod biomass. The results were generalized with linear models for arthropod biomass (49 sampling campaigns over 3 years, including 588 samples) using cropping variant categories on winter wheat. The displayed values are based on comparisons between organic, conventional and NOcsPS cropping variants. Significance was tested by multiple comparisons (Tukey-Kramer test) using SAS. The middle line represents median values. The upper and lower lines represent the first and third quartiles. The lower and upper hinges represent maximum and minimum values excluding suspected outliers. For graphical reasons, extreme outliers are not shown. “+” represents the mean value

In the yellow trap collections, the average biomass was 1.5 g in the organic cropping variant, 1.4 g in the NOcsPS cropping variant and 0.9 g in the conventional cropping variant. In eclector trap captures, the highest arthropod biomass was obtained in the NOcsPS cropping variant, with an average of 3.6 g, which was closely followed by the organic cropping variant with 3.3 g. The conventional cropping variant performed worst with a mean of 2.7 g. The same trend was determined in the sweeping net catches: 1.3 g average arthropod biomass in the NOcsPS cropping variant, 1.1 g in the organic cropping variant and 0.7 g in the conventional cropping variant. In summary, the eclector traps caught the highest arthropod biomass (Fig. 2).

Using a linear model and Tukey post hoc test, there was no statistically significant difference in weighed arthropod biomass of the eclector trap catches between the NOcsPS and the conventional cropping variants (*p*=0.0916). Neither to NOcsPS nor to the conventional cropping variant, the organic cropping variant revealed statistically significant differences (*p*=0.6397 and *p*=0.4548, respectively). In contrast, the yellow trap catching method showed a difference between organic and conventional cropping variants (*p*=0.014), but neither between organic and NOcsPS cropping variants (*p*=0.792) nor between conventional and NOcsPS cropping variants (*p*=0.071). The sweeping net catches were significantly different (*p*=0.001) between NOcsPS and conventional cropping variants, and between organic and conventional cropping variants (*p*=0.043). No significant difference was determined between NOcsPS cropping variant and organic cropping variant (*p*=0.420) (Supplementary Table S2).

### Arthropod abundance

To determine arthropod abundance, the average number of individuals per catching method was calculated in the different cropping variants. In total, 124,215 arthropods were collected with all three catching methods, including 121,143 insects and 3,072 arachnids (Table 1). Consequently, insects comprised 97.5 % of all arthropods collected in this study so that further analyses were only made for arthropods in general.

**Fig. 3:**
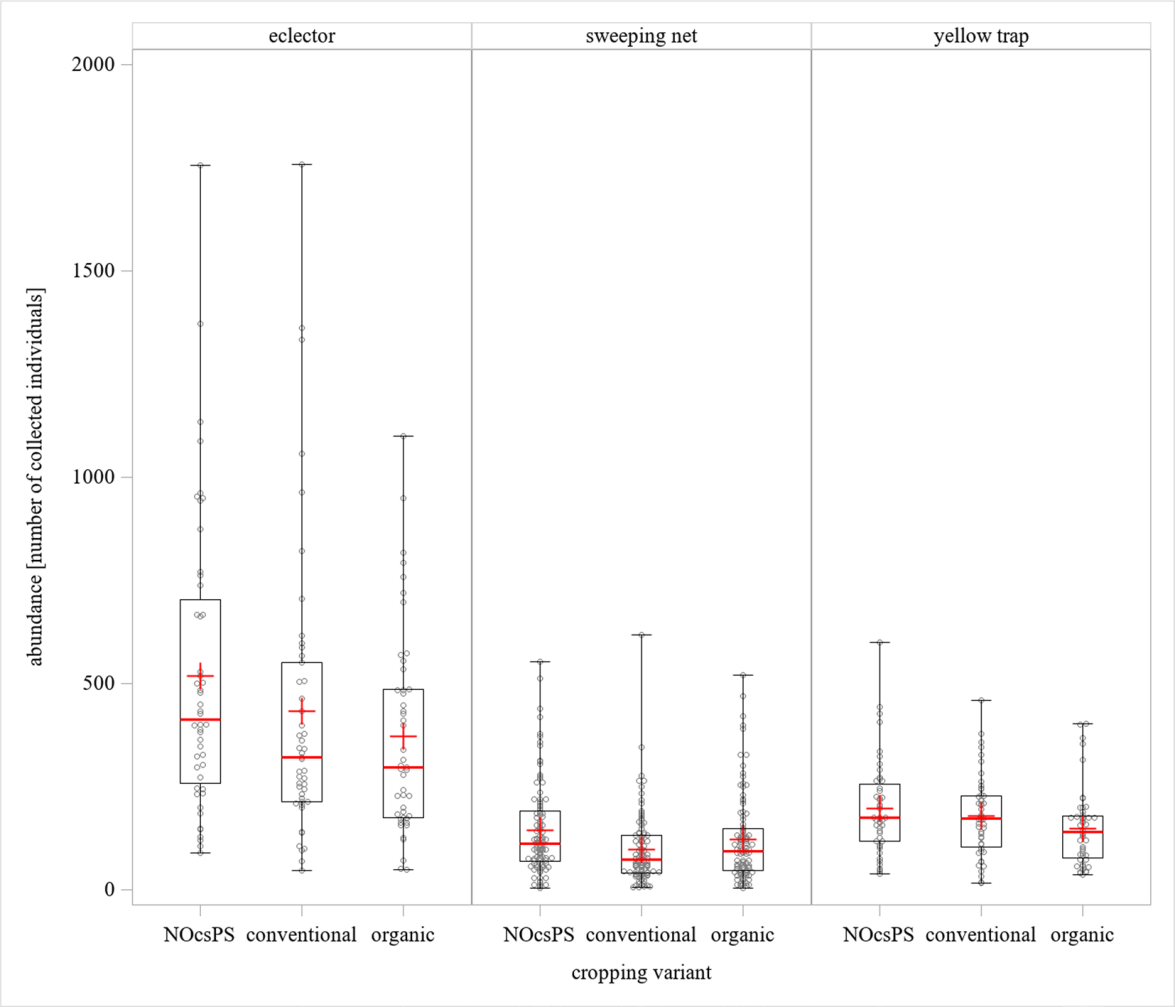
Partial effects of cropping variants on arthropod abundance as determined with the three collection methods. The results were generalized with linear models for arthropod abundance (49 sampling campaigns over 3 years, including 588 samples) using cropping variants categories on winter wheat. The displayed values are based on comparisons between organic, conventional, and NOcsPS cropping variants. Significance was tested by multiple comparisons (Tukey-Kramer test) using SAS. The red lines represent median values. The upper and lower lines represent the first and third quartiles. The lower and upper hinges represent maximum and minimum values excluding suspected outliers. For graphical reasons, extreme outliers are not shown. “+” represents the mean value

The NOcsPS cropping variant showed the highest number of arthropods over the three years. Eclector trap displayed an average of 518.6 individuals whereas the sweeping net method collected an average of 144.6 individuals and the yellow traps an average of 196.6 individuals. In the conventional cropping variant, an average of 432.2 arthropods were caught in eclector traps. The sweeping net catches showed an average of 97 individuals and yellow traps an average of 179 individuals. In the organic cropping variant, an average of 371.9 individuals was collected with the eclector method and averages of 121.8 and 148.7 individuals were obtained with sweeping net and yellow traps, respectively (Fig. 3).

Using a generalized linear model with Poisson distribution of the abundance and Tukey post hoc test, statistical differences regarding the cropping variants were found with every catching method. Eclector traps showed statistical differences in arthropod abundances between organic and conventional cropping variants (*p*<0.0001), organic and NOcsPS cropping variants (*p*<0.0001), and NOcsPS and conventional cropping variants (*p*<0.0001). Statistically significant differences were also existent for the other two trapping methods. Again, all cropping variants showed a statistically significant differences in arthropod abundance of *p*<0.0001 between each other (Supplementary Table S3).

### Relationships between arthropod biomass and arthropod abundance

To examine whether there was a correlation of arthropod biomass with arthropod abundance, a statistical correlation analysis of the two parameters was performed. With respect to the eclector traps, a strong positive correlation was found in the organic cropping variant (r(47)= 0.879, *p*<0.001). The higher the value for the number of individuals was, the higher became the arthropod biomass and vice versa. In conventional and NOcsPS cropping variants, there was also a strong correlation between these two factors (r(48)= 0.801, *p*<0.001 and r(47)=0.854, *p*<0.001). Likewise, a strong, same-directed correlation occurred in all cropping variants in the sweeping net catches (organic: r(100)=0.656, *p*<0.001; conventional: r(100)=0.671, *p*<0.001; NOcsPS: r(100)=0.696, *p*<0.001).

**Fig. 4:**
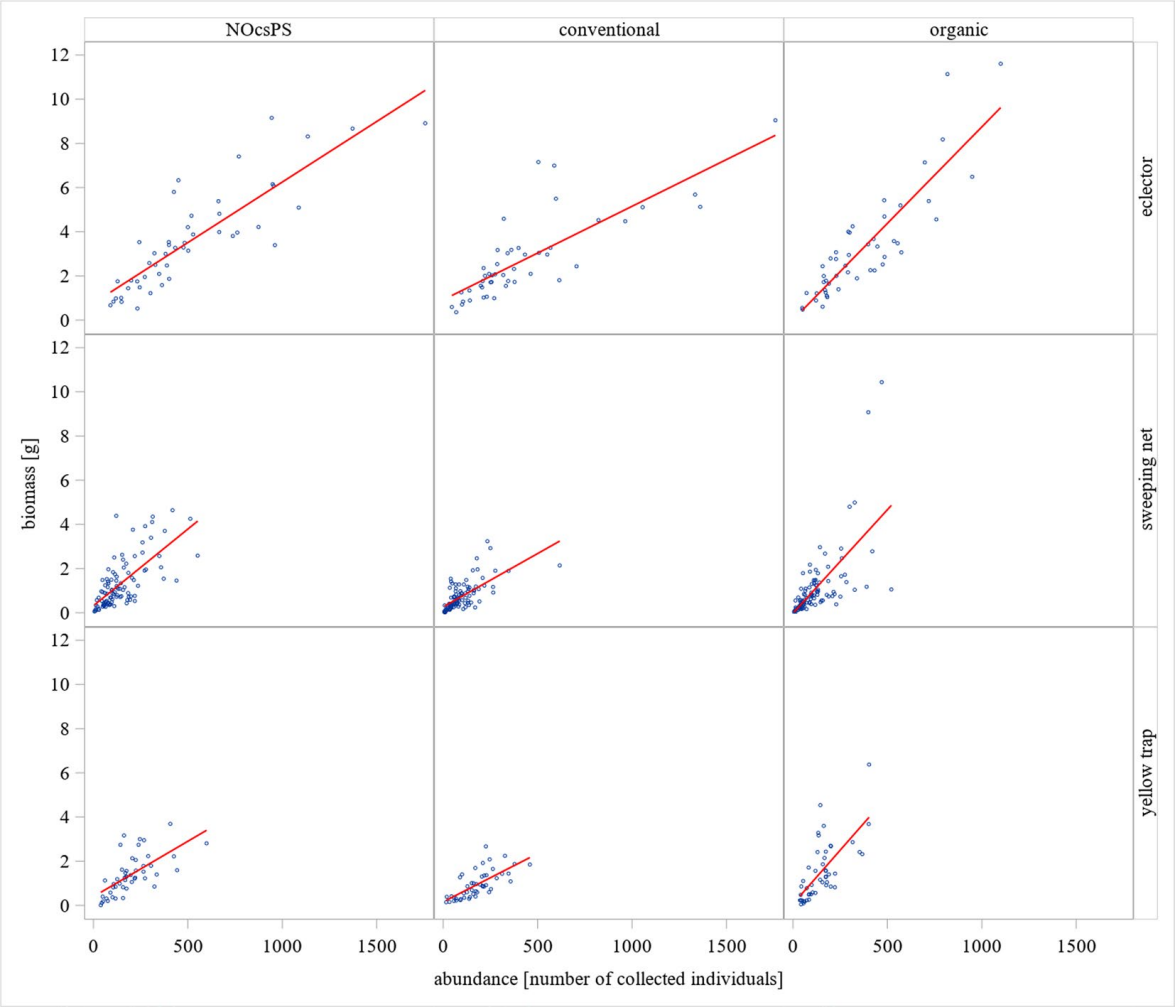
Correlation between arthropod biomass and abundance including linear regression functions

The results obtained with the yellow traps support those of the other two trapping methods. In the organic cropping variant, there is a strong significant correlation between arthropod biomass and arthropod abundance: r(48)=0.692, *p*<0.001. Furthermore, significant strong correlations between these two parameters became evident in the conventional (r(48)=0.698, *p*<0.001) and NOcsPS cropping variants (r(48)=0.647, *p*<0.001). The results therefore suggest that there was a positive, strong correlation between arthropod biomass and arthropod abundance in all three catching methods. An increase in abundance also led to an increase in arthropod biomass and vice versa. The results of the correlation analysis are illustrated in Fig. 4.

### Arthropod richness

Employing a generalized linear model with a Poisson distribution to analyze arthropod richness on order level, along with a Tukey post hoc test, yielded no statistically significant disparities among the cropping variants across all catching methods. The results are outlined in Table 2.

**Table 2:** Arthropod richness comparison between cropping variants using a generalized linear model with Poisson distribution and Tukey post -hoc -test (p ≤ 0.05). The F, df, and p-values are provided for the predictors. For pairwise comparisons (Tukey test), the p-value is reported

Predictors	Collection method					
	Eclector traps		Sweeping net		Yellow trap	
	F(df1,df2)	p value	F(df1,df2)	p value	F(df1,df2)	p value
Collection day	F(2,137)=0.7	0.4986	F(3,294)=5.32	0.0014	F(1,140)=0.27	0.6042
Cropping variant	F(2,137)=0.05	0.9496	F(2,294)=0.76	0.4681	F(2,140)=0.03	0.9656
Tukey test						
NOcsPS vs. conventional		0.9736		0.4652		0.9783
NOcsPS vs. organic		0.9474		0.6521		0.9986
Conventional vs. organic		0.9953		0.9517		0.9662

### Factors influencing arthropod biomass, abundance, and richness

**Fig. 5:**
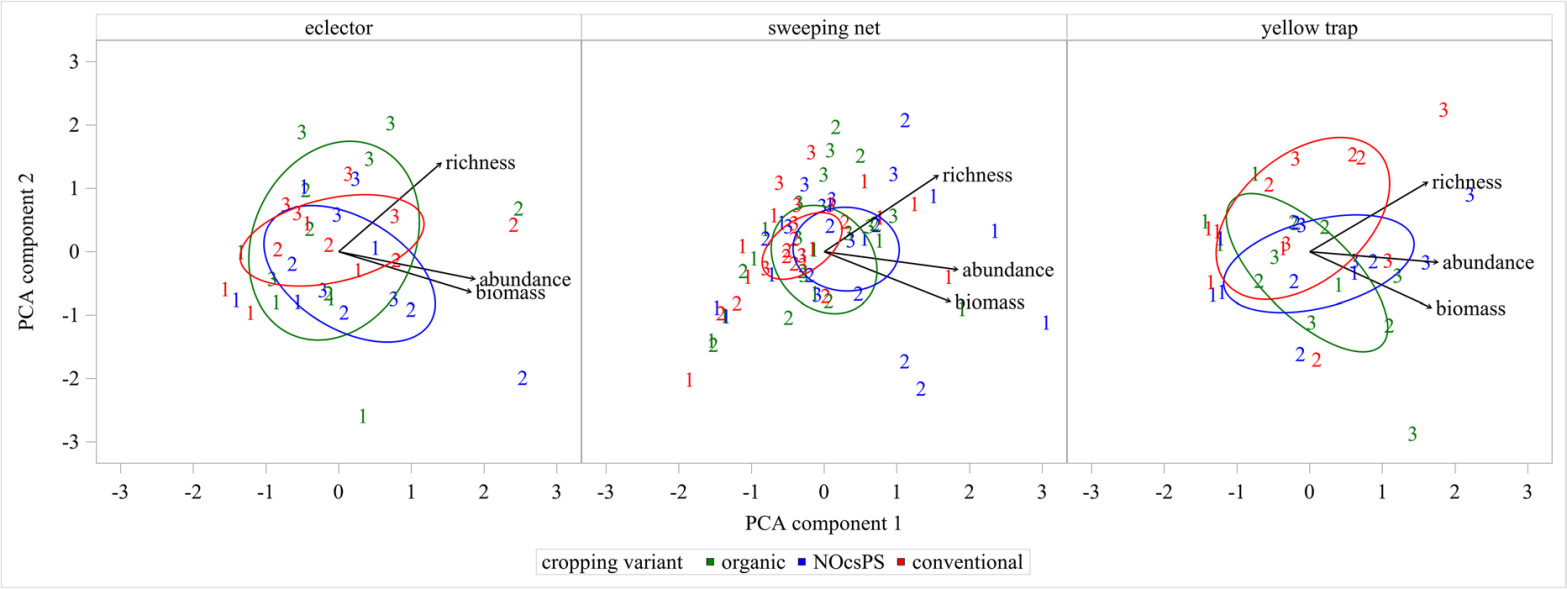
Principal component analysis (PCA)−bi-plot of arthropod biomass, abundance, and richness based on the variance in the cropping variants organic, NOcsPS, and conventional for the catching methods eclector, sweeping net and yellow trap over a 3-year investigation (1 = 2020, 2 = 2021, 3 = 2022). The first two components explained 21.58% and 74.12% of the variances in eclector trap collections, 18.18% and 74.54% of the variances in sweeping net collections and 16.98% and 71.99% of the variances in yellow trap collections, respectively. Arrows indicate the strength of the trait influence on the first two PCs

The outcomes of the PCA fail to elucidate a discernible trend regarding the factors impacting arthropod biomass, abundance, and richness. The extensive range of points in the coordinate system indicates that the influence of those factors extends beyond the cropping variant, notably stemming from temporal variations and suggesting a significant impact of weather conditions (Fig. 5). Similar to the findings of the correlation analyses, the PCA reveals a consistent correlation between arthropod biomass and abundance across all three catching methods. Additionally, a correlation between arthropod biomass and richness could be evidenced at the order level: eclector: r(36)= 0.43009, *p*=0.0088; sweeping net: r(75)= 0.47664, *p*<.0001, yellow trap: r(36)= 0.49472, *p*=0.0022. Furthermore, significant correlations between the two parameters arthropod abundance and arthropod richness became evident (eclector; r(36)= 0.50579, *p*=0.0016; sweeping net: r(75)=0.60542, *p*<.0001; yellow trap: r(36)= 0.59609, *p*=0.0001).

## Discussion

In this study, we aim to analyze and compare the arthropod biomass, abundance, and richness in various cropping systems, including conventional, organic, and a new hybrid cropping variant NOcsPS. To assess the impact of absence of chemical-synthetic pesticides on arthropod presence within these wheat cropping systems, we used three different catching methods. By investigating the differences in arthropod populations among these cropping variants, we expected to gain valuable insights into the effects of different agricultural practices on arthropod communities and ecosystem health.

The analyses of the collected arthropods clearly demonstrate differences in arthropod biomass and abundance between the different cropping variants. Although arthropod biomass varied depending on the catching method, it generally appears to be positively affected by crop management without chemical-synthetic pesticides and adapted nitrogen fertilization.

Interestingly, the NOcsPS cropping variant exhibited the highest arthropod abundance over the three study years and the highest arthropod biomass in two of the three collecting methods, indicating a more diverse and abundant arthropod community than the conventional and organic cropping systems. The conventional cropping variant showed lower arthropod biomass compared to both organic and NOcsPS management systems. However, it exhibited superior results in terms of arthropod abundance than the organic cropping variant with two catching methods. The latter demonstrated moderate levels of arthropod biomass, suggesting a balanced ecological impact as compared to the other two cropping variants, but presented lower arthropod abundances.

Our results confirm previous studies on insect diversity and abundance in differently intensive cropping systems (Bengtsson et al., 2005; Sanders & Heß, 2019). Those studies compared species numbers and insect abundance, but it was assumed that higher insect abundance was associated with higher biomass.

Several factors can influence the biomass and abundance of arthropods in agriculturally landscapes. One important factor is the application of pesticides. In conventional farming, the use of pesticides can lead to a decrease in arthropod abundance due to direct toxicity and disturbance of natural habitats and food sources. On the other hand, organic farming, which avoids chemical-synthetic pesticides and uses only organic fertilizers, usually brings forth a higher biodiversity than conventional farming and can create a more favorable environment for arthropods, leading to a higher biomass and abundance (Bengtsson et al., 2005; Freier et al., 2017; Stein-Bachinger et al., 2021).

An insecticide containing the active ingredient lambda-cyhalothrin was applied in the conventional cropping variant to control the larvae of ground beetle in 2021 in repetitions 1 and 4 and in 2022 in repetition 2. This synthetic pyrethroid causes strong feeding and contact effects, which starts very quickly after application. It cannot be excluded that the application affected non-target organisms and therefore resulted in lower arthropod abundance in this cropping variant. The disruption by application lambda-cyhalothrin of insect populations serving as food sources for natural predators may indirectly lead to a decline in predator populations, too. The insecticide is also effective against aphids and may have affected natural antagonists of aphids such as Coccinellidae and Chrysopidae directly and indirectly by causing increased mortality and decreased fertility rates as well as reduced oviposition rates and avoidance behavior towards treated plants (Luna et al., 2018; Mills et al., 2016; Spíndola et al., 2013). The use of insecticides against the occurrence of aphids and the resulting reduced aphid abundance (as shown in Supplementary Table S4) deprives aphid antagonists of their food source. As a consequence, numbers of both larval and adult ladybugs, hoverflies, and lacewings decrease (Geiger et al., 2009).

While our findings revealed no statistically significant differences in arthropod richness at the order level, and detailed species richness analyses were not feasible, our study did indicate certain trends within various arthropod orders and families. The overall average of the three years showed that the highest number of arachnids was caught in NOcsPS with all three catching methods. The conventional cropping variant showed higher numbers of collected arachnids with the sweeping net catches and the yellow traps than the organic cropping variant, supporting previous studies demonstrating that not all arthropod taxa benefit from organic farming (Birkhofer et al., 2014), where a positive effect was mainly visible on phytophagous species while predators were disadvantaged. Belonging to the latter group, spiders and ground beetles even achieved higher individual abundances and species richness in conventional farming systems as compared to organic farming. Results from previous studies (Geiger et al., 2009; Birkhofer et al., 2012, Birkhofer et al., 2013) could not be repeated here.

The order Diptera seems to benefit most from the NOcsPS cropping variant and showed lower abundance in the organic than in the conventional cropping variant. Diptera fulfil significant ecological functions and possess substantial human relevance, e.g., serving as important pollinators, thus contributing significantly to ecosystem services and agricultural productivity. Further analysis revealed that certain arthropod groups, such as pollinators and predators, were more prevalent in the organic and NOcsPS cropping variant than in the conventional cropping variant (Supplementary Table S4). This highlights the potential ecological benefits of the first two management systems in promoting biodiversity and ecosystem resilience.

Upon a closer look at the family of Apoidea, it becomes clear, that abundance decreased with the intensification of the cropping system (in terms of pesticides and fertilization) in all catching methods. This trend supports the findings of Sponsler et al. (2019), who claimed that all pesticide-pollinator interactions are causally downstream from pesticide use, when pesticides exert sublethal and lethal effects on pollinators. These effects do not only result from insecticides but also from herbicide and fungicide application. The abundance of other pollinator families, such as Syrphidae, did not show such a clear trend in our study, but rather fluctuations between the study years and the collecting methods.

Differences in the abundance of arthropod taxa between the cropping variants could also be attributed to their habitat or plant preferences. Diversified habitats found in organic and NOcsPS cropping variant offer arthropods a greater variety of food sources and shelters in form of weed, leading to higher arthropod biomass and abundance as compared to the conventional cropping variant with less diverse habitats. Additionally, the presence of flowering plants and weed in organic and NOcsPS cropping variants can support arthropod populations by providing nectar and pollen. The loss of weed diversity and food resource resulting from herbicide application in the conventional cropping variant can decrease populations of pollinators and natural predators of plant pests. The absence of flowering weeds in the conventional cropping variant may lead to reduced abundance of wild bees and hoverflies. Both groups of pollinators use flowering plants as a food source and are often linked to specific plant species, which may lead to the disappearance of the pollinator following the disappearance of the plants (Biesmeijer et al., 2006). It has been verified that the composition of the hoverfly community is significantly influenced by agricultural intensity and the availability of floral resources (Lucas et al., 2017). Furthermore, a few studies indicate that there is a direct impact of herbicides on oviposition and hatching rates, larval growth rates, periods and survival, and mortality of certain insect species (Sharma et al., 2018). Moreover, the use of herbicides leads to the destruction of overwintering habitats of arthropods and thus to a lower abundance in the following year (Sotherton, 1984). The reduction of hosts, shelter, and food resources in the form of flowering weeds in agricultural fields impacts the pollinator occurrence and should be compensated for (Rands & Whitney, 2011). The utilization of fungicidal agents targeting pathogens such as *Septoria tritici* within the conventional cropping variant may exert indirect influences on arthropod populations, especially pollinators as bees, even though only a few studies dealt with this problem (Cullen et al., 2019; Desneux et al., 2007; Ladurner et al., 2005).

Another critical variable examined in our study pertains to the impact of mineral nitrogen application, characteristic of the new NOcsPS cropping system, on the prevalence of arthropod species. While in organic farming, the soil is enriched with nitrogen by crop rotation (e.g., lupin clover grass) or dung, liquid manure, and slurry, mineral fertilizers are used to enhance the yield of agricultural crops in conventional farming. Nitrogen not only has a direct beneficial effect on the population and larval development of insects and the number of predators, but also acts indirectly by strengthening the defense mechanisms of plants and influencing the composition of flowering weeds, which are food sources for pollinators and aphid antagonists (Chen et al., 2008; Olson et al., 2009; Veromann et al., 2013).

Our analysis revealed a positive correlation between arthropod abundance and biomass in all catching methods, indicating that areas with higher arthropod abundance also tended to have greater biomass. This suggests that the abundance of arthropods is closely linked to the overall biomass of the ecosystem, highlighting the importance of arthropods in supporting ecosystem health and functioning. These findings emphasize the need for conservation strategies that prioritize the protection and promotion of arthropod populations to ensure the resilience and stability of ecosystems. Other studies concluded that insect biomass is not necessarily informative of insect biodiversity (Homburg et al., 2019; Saint-Germain et al., 2007). One potential explanation is that the reduction in the number of species may be compensated for by an increase in the number of individuals from other species, or few larger and heavier insects may be replaced by a higher number of smaller and lighter ones (Heleno et al., 2009; Shortall et al.; 2009).

Our results presented on arthropod biomass and abundance are helpful to evaluate differences in wheat ecosystems and ecosystem management. These two parameters should not be used individually but complement each other to get a better overview of arthropod occurrence in winter wheat. They are, however, not appropriate to draw conclusions on species richness which should be evaluated as well from an ecological point of view when deciding for or against a cropping system. Ideally, the arthropod collections of such a study should therefore be identified to species level and probably be carried out over several cropping seasons to compensate for seasonal fluctuations.

It is often criticized that the price for the positive effects of organic farming on biodiversity is high yield losses (Gabriel et al., 2013). Especially in times of climate change, it is therefore important to consider how these yield losses can be minimized. The supplementary application of nitrogen in mineral form may serve to meet crop demands efficiently, thereby ensuring consistent yields, while concurrently fostering a beneficial impact on biodiversity through the reduction of pesticides. In the present study, only a 25% yield loss (mean 2020-2022: 6.47 t/ha) occurred in the NOcsPS cropping variant due to the use of mineral fertilization compared to the yield in the conventional cropping variant (8.55 t/ha), while the organic cropping variant had a 43% yield loss (mean 4.85 t/ha) compared to the conventional cropping variant. Overall, the combination of avoiding pesticides with simultaneous adapted nitrogen fertilization seems to have positive effects on the arthropod biomass and abundance in general, and on the arthropod richness of some arthropod families including important pollinators. The present study underscores the importance of considering arthropod biomass and abundance when evaluating the sustainability and ecological impact of different agricultural practices. The findings contribute to our understanding of how crop management strategies can influence arthropod communities and ultimately ecosystem health. By incorporating these insights into agricultural decision-making processes, we can work towards fostering more sustainable and ecologically resilient farming systems.

## Conclusion

The new hybrid NOcsPS cropping variant turned out to be the system with the highest values regarding arthropod abundance in every collecting method and the highest arthropod biomass as obtained with two of the three analyzed trapping methods. This demonstrates that the absence of chemical-synthetic pesticides and adapted nitrogen fertilization supports arthropod occurrence. A cultivation system that does not permit the use of chemical-synthetic pesticides, but allows the targeted use of mineral fertilizers, could therefore be appropriate to complement existing cultivation systems and thus represent an agricultural system of the future. Particularly in regions where organic farming is difficult to implement (e.g., in arid areas), this system offers a possibility of environmentally friendly and biodiversity-preserving farming. The NOcsPS cropping variant could compensate for the shortcomings of organic farming in terms of harvest quantities and qualities and thus supplement organic farming instead of replacing it.

To comprehensively discuss the environmental advantages of employing the new cropping variant, further research is necessary across various crops and environmental contexts, as well as enduring impacts of NOcsPS strategies across varied agricultural environments, incorporating more extensive taxonomic analyses of arthropod species. Given our observation that non-chemical pest control strategies enhance both arthropod biomass and abundance, forthcoming research could delve into elucidating the precise mechanisms underpinning this phenomenon, assessing the long-term viability of these practices, or investigating their implications for crop yields and soil health.

### Supplementary Information

Below is the link to the electronic supplementary material.

Supplementary file1 Table S1: Field books of the NOcsPS experimental field. List of plant cultivation measures for the trial years 2020-2022. (XLSX 33 KB)

Supplementary file2 Table S2: Arthropod biomass comparison between cropping variants using a linear model and Tukey post-hoc-test (p ≤ 0.05). F, df and p-values are provided for the predictors. For pairwise comparisons (Tukey test), the p-value is reported. As key figures for the model, R² (coefficient of determination) and the p-value of the F-test are reported. The F-test is used to examine whether at least one of the predictors in the model was significant. (XLSX 18 KB)

Supplementary file3 Table S3: Arthropod abundance comparison between cropping variants using a generalized linear model with Poisson distribution of the abundance and Tukey post-hoc-test (p ≤ 0.05). The F, df and p-values are provided for the predictors. For pairwise comparisons (Tukey test), the p-value is reported. As key figures for the model, R² (the coefficient of determination) and the p-value of the F-test are reported. The F-test is used to examine whether at least one of the predictors in the model is significant. (XLSX 18 KB)

Supplementary file4 Table S4: List of collected arthropods classified according to taxonomic categories, depending on study year and cropping variant. (XLSX 27 KB)

## Data Availability

Data supporting the conclusions of this article are included within the article and its Supplementary Tables.
